# Determinants of modern contraceptive practice in Yaoundé-Cameroon: a community based cross sectional study

**DOI:** 10.1186/s13104-017-2543-7

**Published:** 2017-06-24

**Authors:** Philip Nana Njotang, Martin Ndinakie Yakum, Atem Bethel Ajong, Marie José Essi, Ebile Walter Akoh, Nzene Edmond Mesumbe, Simon Ako, Enow Robinson Mbu

**Affiliations:** 1 Department of Obstetrics and Gynaecology, Faculty of Medicine and Biomedical Sciences, University of Yaoundé I, Yaoundé, Cameroon; 2 Obstetrics and Gynaecology Unit, Yaoundé Central Hospital, Yaoundé, Cameroon; 3 Department of Public Health, Faculty of Medicine and Biomedical Sciences, University of Yaoundé I, Yaoundé, Cameroon; 4 Meilleur accès aux soins de Santé, Yaoundé, Cameroon; 5 Directorate of Family Health, Ministry of Public Health, Yaoundé, Cameroon

**Keywords:** Contraceptive-use, Modern-contraception, Factors, Child-bearing-age, Yaoundé, Cameroon

## Abstract

**Background:**

Despite numerous efforts put in place to increase modern contraceptive use in Cameroon as a means to fight maternal and infant mortality, the prevalence of modern contraception has shown only a slow increase and maternal mortality is constantly rising. This paper attempts to identify barriers to contraceptive use in Biyem-Assi, Yaoundé-Cameroon so as to clearly define in which domain and how to intervene concerning contraceptive use in Cameroon.

**Methods:**

It was a community-based cross sectional study involving a two-steps cluster sampling. Data were collected from November 2014 to April 2015 and analysis done with Epi-Info version 3.5.4. Association between contraceptive use and independent factors was estimated by calculating odds ratio (OR) and confidence interval at 95%. Significance of association in univariate analysis was estimated by calculating the p value with chi2 test. Potential confounder (pregnancy intention) controlled in a multiple logistic regression.

**Results:**

A total of 613 sexually active women were enrolled into the study with a mean age of 27.2 (δ ± 6.2) years. Among the women, 293 (47.8%) were in a union and 530 (86.8%) of them had attended at least a secondary education. Also, 107 (17.5%) responded that their beliefs do not approve contraceptive use and 101 (16.6%) said their partners do not approve contraception. At the moment of data collection, 361 (58.9 [54.9–62.8] %) were currently using a modern contraceptive method. The rate of use of modern contraception was significantly lower in women in a union (OR 0.57, p = 0.0002) and in those with age greater than 30 years (OR 0.45, p = 0.0004). Conversely, the rate of use was significantly higher in women whose partners approved contraception (OR 4.14, p = 0.0000) or when family planning was discussed within the couple (OR 1.93, p = 0.0028).

**Conclusion:**

The rate of use of modern contraception in Biyem-Assi Health District is relatively high. Women in a union and those aged greater than 30 years turn to be less likely to use a contraceptive method than the rest of the population meanwhile women whose partner approve contraceptive-use or who discuss about family planning with their partners, are most likely to use a contraceptive method than others. To increase the rate of use of modern contraception in Yaoundé-Cameroon, interventions should target more of couples and not women alone.

## Background

Family planning allows individuals and couples to anticipate and attain their desired number of children while helping them to space and limit births. It is achieved through the use of contraceptive methods [1, 2]. A woman’s ability to space her pregnancies has a direct impact on her health and wellbeing as well as on the outcome of pregnancy. The use of modern contraceptive methods is one of the cornerstones in the fight against maternal, infant and neonatal morbi-mortality. It reduces the need for abortion, especially unsafe abortion [1, 2]. Though Africa hosts only 12% of the world’s population, 99% of maternal deaths in the World are registered in Africa [3].

In Cameroon, the rate of maternal mortality is very high, with a good proportion attributed to unsafe abortion [4–7]. A majority of abortions in Cameroon are induced; most resulting from unplanned pregnancies [2]. In 2013, an estimated 40% of pregnancies in Cameroon were unintended [2]. Cameroon therefore is called to step-up the use of modern contraception in order to take control of this rising family health threat.

The results of recent demographic and health surveys show that the rate of use of modern contraception among women in a union has gradually risen in Cameroon from 1991 to 2011 [8]. Despite this increase, the rate has slightly dropped in Yaoundé and Douala between 2004 and 2011 [8]. The consequence of not using contraception is very heavy on Cameroon. In fact, it was documented in 2013 that Cameroon can save about 2.7 billion XAF every year by providing half of the contraception needs in the country meanwhile about 6000 women die every year in Cameroon due to pregnancy and child bearing because of a lack of access to modern services of family planning [1].

It is now clear that increasing contraceptive use in Cameroon requires an insight of the determinants of use and non-use of contraception. This can permit the redesigning of existing interventions and designing of new ones to better tackle this component of family planning before it slips up. The use of modern contraceptive methods can be influenced by many factors which can be grouped into factors related to the health system and factors related to the woman and her environments [9–13]. This paper attempts to identify barriers to the modern contraceptive use in the Biyem-Assi Health district in Cameroon by looking critically at factors related to the woman and her environments.

## Methods

### Consent to participant

Before the administration of questionnaire, participants were administered an information notice and informed consent signed for those who accepted to participate. For respondents less than 18 years of age, the consent of their legal representatives was obtained and an assent from each of the respondent. The study was approved by the Biyem-Assi District Health service.

### Study design

It was a cross-sectional community based study targeting women of child-bearing-age (15–49 years) in the Biyem-Assi health district conducted from November 2014 to April 2015. Data were collected by surveyors with the help of a questionnaire administered face-to-face. A two-steps cluster sampling was used to select participants and data was analysis with EpiInfo version 3.5.4. The strength of association was measured by calculating the odds ratio (OR) and the significance by estimating the p value. Potential confounder (pregnancy intention) was controlled in a multiple logistic regression.

### Study population

The study targeted women aged between 15 and 49 years in the Biyem-Assi Health District. However, visitors to the district, pregnant women, women who were menopausal/hysterectomized, sub-fecund women, postpartum amenorrheic women, and women whose last sexual contact was more than 3 months to the study time were excluded.

### Study settings

Biyem-Assi Health District is found in the Centre region of Cameroon and forms part of the 6 health districts of Yaoundé. It is the most populated health district in Yaoundé and has an estimated population of about 334,333 people for 2014 unevenly distributed over 4 health areas. It has a total of 94 health facilities including the university teaching hospital of Yaoundé. The study site was chosen because Yaoundé had the highest prevalence of contraceptive use in 2011 but with the rate of use reduced recently and Biyem-Assi is the largest and most populated district of Yaoundé. The choice was equally influenced by the population composition which is very diverse with all the regions of Cameroon represented.

### Sampling technique

The population of Biyem-Assi was divided into 70 geographical zones (clusters) and 50 of them were selected by a simple random sampling with replacement. In each selected zone, major road junctions (points where 2 major roads meet) were identified and one selected randomly for a starting point. The direction to follow in a chosen junction was selected by tossing a plastic bottle and following the direction pointed by the head. Households were enrolled only on the left hand side of the surveyors. All household on the left hand side were included until the desire number of participants was attained. All eligible participants in a selected household were enrolled for the study.

### Definition of operational terms

- *A woman in a consensual union:* a woman living with the partner under the same roof as married without formal ratification by the law for at least six (6) months.
- *Single:* a woman who is sexually active and not in a consensual union.
- *Modern contraceptive methods:* male and female condoms, intrauterine device, pills, subcutaneous implants, injectable contraceptives, diaphragms, cervical caps.
- *Current contraceptive user:* a participant who was currently using a contraceptive method.

### Data collection

Data were collected by trained surveyors who were rigorously supervised by trained field supervisors. Once in a selected household, the surveyor after the approval of the head of the household, screened to identify eligible women. Each eligible woman after the administration of the information notice was invited to participate in the study. For those who accepted, an informed consent was signed and the questionnaire administered. The respondents were interviewed in strictly confidentiality in which only the surveyor and the respondent were involved without the hearing of a third party whosoever. The questionnaire was readout to the participants considering the low level of education that some of them might had. Data were collected on socio-demographic information of the participants and partner; the use of contraception; contraceptive preferences as well as their fertility intension evaluated.

### Data management

Data forms were checked, validated, coded, double-entered into the computer, and compared for quality control. Data entering and analysis were done with EpiInfo version 3.5.4. Socio-demographic data were analyzed by running frequency for categorical variables like marital status and by calculating means for continuous variables like age. Association between contraceptive use and independent factors like, level of education, partner’s decision on contraception, number of children alive etc. was estimated by calculating the OR and the confidence interval at 95% and significant of the association in univariate analysis was measured by estimating the p value using chi2 test (we were dealing with bimodal categorical variables with expected outcomes per cell in 2 × 2 contingency table all above 5). Participants planning to get pregnant within the next 2 years were controlled in a multiple logistic regression as a potential confounder.

## Results

### Socio-demographic information

A total of 613 sexually active women were enrolled into the study with a mean age of about 27.2 (δ ± 6.2) years. Table 1 shows the some baseline characteristics of the participants and it can be noted there that 293 (47.8%) were in a union, that is, either 167 (27.2%) legally married and 126 (20.6%) consensual union. Also, more than 90% of participants had at least a child already at the moment of data collection. Concerning the level of education, 530 (86.8%) of them had acquired at least a secondary education. Regarding their beliefs and partners decision, 107 (17.5%) responded that their beliefs do not approve of contraception and 101 (16.6%) said their partners do not approve contraception.

**Table 1:** Baseline characteristics of participants

Indicator	Modalities	Number	Percentage (%)
Marital status	Married	167	27.2
	Consensual union	126	20.6
	Single	320	52.2
Level of education	Did not school	1	0.2
	Primary	80	13.1
	Secondary	276	45.2
	High school and university	254	41.6
Level of education of the partner	Do not know	7	1.2
	Did not school	4	0.7
	Primary	64	10.6
	Secondary	216	35.9
	High school and university	311	51.7
Number of living children	0	36	8.9
	1	173	42.6
	2	94	23.2
	3	51	12.6
	>3	52	12.7
Does your belief accept contraceptive use?	Yes	107	17.5
Does your partner approve contraceptive use?	Yes	101	16.6

### Modern contraceptive use and factors associated

At the moment of data collection, 424 (69.6%) of the women were using a contraceptive method (either traditional or modern) and 361 (58.9 [54.9–62.8] %) were currently using a modern contraceptive methods (modern contraceptive prevalence). Among those who were not presently using a contraceptive method, 10 (4.0%) said they were afraid of the side effects. Table 2 presents the prevalence of use of modern contraceptive methods stratified by the marital status of the women.

**Table 2:** Prevalence modern contraception stratified by marital status

	Marital status (%)			
	Single (n = 314)	Consensual union (n = 126)	Married (n = 167)	Divorced (n = 6)
Prevalence of use of modern contraception (n = 613)	206 (65.6)	72 (57.1)	78 (46.7)	5 (83.3)

It can be noted from Table 1 that the prevalence of modern contraception is highest in single or divorced women and lowest in married women. Table 3 presents factors associated with modern contraceptive use.

**Table 3:** Factors associated with modern contraceptive use among women of child-bearing age in the Biyem-Assi Health District, Yaoundé-Cameroon

Factors	Univariate analysis			Multivariate analysis		
	OR	CI 95%	p value	OR_Adj_	Adj CI 95%	p value
Woman in a union i.e. married or in a consensual union (yes/no)	0.54	0.3913–0.7503	0.0002*	0.57	0.4099–0.8034	0.0012*
Age greater than 30 years (yes/no)	0.46	0.3127–0.6789	0.0001*	0.45	0.2889–0.6991	0.0004*
Level of education higher than primary (yes/no)	0.99	0.6204–1.5896	0.9769	0.88	0.5405–1.4400	0.6161
Number of children alive >5 (yes/no)	0.26	0.0671–0.9729	0.0455*	0.32	0.0807–1.2663	0.1044
Health personnel as main source of information (Y/N)	1.08	0.7852–1.4970	0.6236	1.09	0.7876–1.5187	0.5931
Do your beliefs accept contraception? (yes/no)	1.25	0.8231–1.9063	0.2930	0.28	0.8342–1.9612	0.2590
Does your partner approve of contraception? (yes/no)	4.00	2.5617–6.2408	0.0000*	4.14	2.6421–6.4853	0.0000*
Discussion of family planning within the couple (yes/no)	2.00	1.3068–3.0529	0.0014*	1.93	1.2543–2.9674	0.0028*
Monthly revenue >100,000 FCFA	0.67	0.4041–1.0964	0.1099	0.66	0.4021–1.0980	0.1107

The threshold of significance is set at p value = 0.05

* Signifies statistically significant

Table 3 shows that the women who were in a union or those aged greater than 30 years were less likely to be using modern contraceptive methods. Also, it indicates that if the partner approves the use of contraception or if the couples discuss about family planning, the woman will be more likely to use a contraceptive method.

## Discussion

This paper aims to identify ‘user-centered’ factors associated with the use of modern contraception in Yaoundé-Cameroon. This can help in the understanding of barriers to contraceptive use and to design evidence-based interventions aimed to increase modern contraceptive use among women in Cameroon.

The results of this study show that at the moment of data collection, 361 (58.9 [54.9–62.8] %) were currently using a modern contraceptive method. The rate of use of modern contraception was significantly lower in women in a union (OR 0.57, p = 0.0002) and in those with age greater than 30 years (OR 0.45, p = 0.0004). Conversely, the rate of use was significantly high in women whose partners approved contraception (OR 4.14, p = 0.0000) or when family planning was discussed within the couple (OR 1.93, p = 0.0028).

The prevalence of modern contraception reported here in Yaoundé-Cameroon is relatively high. However, about 2/5 of the women of child bearing age are still at risk of unwanted pregnancy. This is a general problem and has been reported in several parts of the world especially in developing countries [8–10, 14]. The results in this study have shown a little improvement compared to other publications [8–10, 14]. This improvement could be attributed to the urbanized nature of Biyem-Assi Health District with several health facilities of various grades found almost everywhere. However, 2/5 of the women sexually active were not using a contraceptive method. The reasons for non-use could include, fear of side effects [15], religious and cultural beliefs being against contraceptive use [16, 17], intention to get pregnant in the nearest future [17], and partner’s disapproval of contraception [3, 9]. However, only 8 (3.2%) of those who were not using a contraceptive method declared that they wanted to get pregnant within 1 year. This shows that 40% of the participants did not plan to get pregnant within one year and were not using any contraceptive method; exposing these women to unintended pregnancies which can equally lead to high rate of induced abortion [2, 4, 7, 9, 11]. Only therapeutic induced abortion is accepted by law in Cameroon [17], most of these abortions are therefore done in hiding, and often under poor conditions. Consequently, women going for induced abortion are at a higher risk of complications including maternal death [5, 6, 12].

This study shows that women in a union (married or consensual union) and those aged greater than 30 years were less likely to be using contraception than the rest of the population. Also, women whose partners approved contraceptive use or who discussed about contraceptive use with their partners, were more likely to be using a contraceptive method than others. The influence of men (partners) in contraceptive use among women is universally known and documented [3, 9]. When a woman is married or living with the partner under the same roof, the partner’s opinion and decision on contraceptive use become influential [3]. Many papers have demonstrated this in the form of intimate partner violence [9] while others see it as consensus [3]. Targeting men with family planning interventions could be very helpful to increase contraceptive use in Cameroon. Therefore, sensitisation and counselling activities should focus on couples, men and women.

The shortcomings of this paper include the fact that data was collected only from women and not from their partners; data was collected by interview and the information given by the women could not be verified; no information about the availability, access, knowledge of women on various contraceptive methods was collected. However, the selection of participants was done randomly and surveyor discussed with the participants well during the consenting procedures to ensure a level of confidence and truth telling before administering the questionnaire. Therefore, this paper gives a snapshot of Biyem-Assi Health District in terms of contraceptive use and could be helpful in decision making.

## Conclusion

The prevalence of modern contraception in the Biyem-Assi Health District is relatively high (58.9%). Also, women in a union (married or consensual union) and those aged greater than 30 years turn to be less likely to use a contraceptive method than the rest of the population meanwhile women whose partner approve contraceptive-use or who discuss about contraceptive use with their partners, are most likely to use a contraceptive method than others. This demonstrates the need to involve men in all aspects of family planning activities. We therefore recommend the following: Health personnel should offer family planning counselling equally to both sexually active women and men and couples stressing on the importance of contraceptive use and the different modern methods available; Researchers should assess the knowledge of sexually active men in contraceptive use and the availability of contraceptive methods in the Cameroon health market.

## References

[1] Vlassoff M, Jerman J (2014). Benefits of meeting the contraceptive needs of Cameroonian women. s.l.

[2] International Federation of Gynecology and Obstetrics (2014). Prévention des avortements à risque et de leurs conséquences. Int J Gynecol Obstet.

[3] Vouking MZ, Evina CD, Tadenfok CN (2014). Male involvement in family planning decision making in sub-Saharan Africa—what the evidence suggests. Pan Afr Med J.

[4] Mosoko A, Delvaux T, Glynn JR, Zekeng L, Macauley I, Buve A (2004). Induced abortion among women attending antenatal clinics in Yaounde, Cameroon. East Afr Med J.

[5] Ekane GEH, Obinchemti TE, Tchente CN, Fokunang LK, Njamen TN, Bechem NN, Njie MM, Latum D (2014). Attainment of the fifth millennium development goal: Utopia or reality based on trends in maternal mortality in 12 years in Two Regional Hospitals in Fako Division, Cameroon? A retrospective study. Open J Obstet Gynecol.

[6] Tebeu PM, Ngassa P, Kouam L, Major AL, Fomulu JN (2007). Maternal mortality in Maroua Provincial Hospital, Cameroon (2003–2005). West Indian Med J.

[7] Schuster S (2005). Abortion in the Moral World of the Cameroon Grassfields. Reprod Health Matters.

[8] National Institute of Statistics (2012). Demographic and Health survey and Multiple Indicators Cluster Survey DHS-MICS 2011. s.l.

[9] Dalal K, Andrews J, Dawad S (2011). Contraception use and associations, with intimate partner violence among women in Bangladesh. J Biosoc Sci.

[10] Fusi-Ngwa CK, Payne VK, Asakizi AN, Katte BF (2013). Knowledge and practice of family planning in Dschang municipality, Cameroon. Afr J Reprod Health.

[11] Nkwabong E, Mbu RE, Fomulu JN (2014). How risky are second trimester clandestine abortions in Cameroon: a retrospective descriptive study. BMC Women’s Health.

[12] Elie N, Efuetnkeng B, Nelson FJ (2014). Outcome of clandestine abortions in two University Teaching Hospitals in Yaoundé, Cameroon. Health Sci Dis.

[13] Mah Mungyeh E, Chiabi A, Tchokoteu Pouasse FL, Nguefack S, Bogne JB, Siyou H, Soh Fru F, Mbonda E, Tchokoteu PF (2014). Neonatal mortality in a referral hospital in Cameroon over a seven year period: trends, associated factors and causes. Afr Health Sci.

[14] Egede JO, Onoh RC, Umeora OU, Iyoke CA, Dimejesi IB, Lawani LO (2015). Contraceptive prevalence and preference in a cohort of south–east Nigerian women. Dovepress.

[15] Cynthia A (2002). Graham: sexual side effects of oral contraceptives: clinical considerations. Med Aspect Hum Sex.

[16] Mankaa W, Kollo B, Doh A (2005). Knowledge, attitudes and practice of contraception amongst secondary school students in Yaoundé, Cameroon: a study of perception differences between males and females. Clin Mother Child Health.

[17] Ministry of Health (2006). National program on reproductive health 2005–2010.

